# Supplementary material for: Distal axotomy enhances retrograde presynaptic excitability onto injured pyramidal neurons via trans-synaptic signaling
Source: Nat Commun. 2017 Sep 20;8:625. doi: 10.1038/s41467-017-00652-y (PMC5607003; doi:10.1038/s41467-017-00652-y)
Supplement: Supplementary file 1 — Supplementary Information [file 41467_2017_652_MOESM1_ESM.pdf]

Description of Supplementary Files

File Name: Supplementary Information
Description: Supplementary Figures, and Supplementary Tables

File Name: Peer Review File

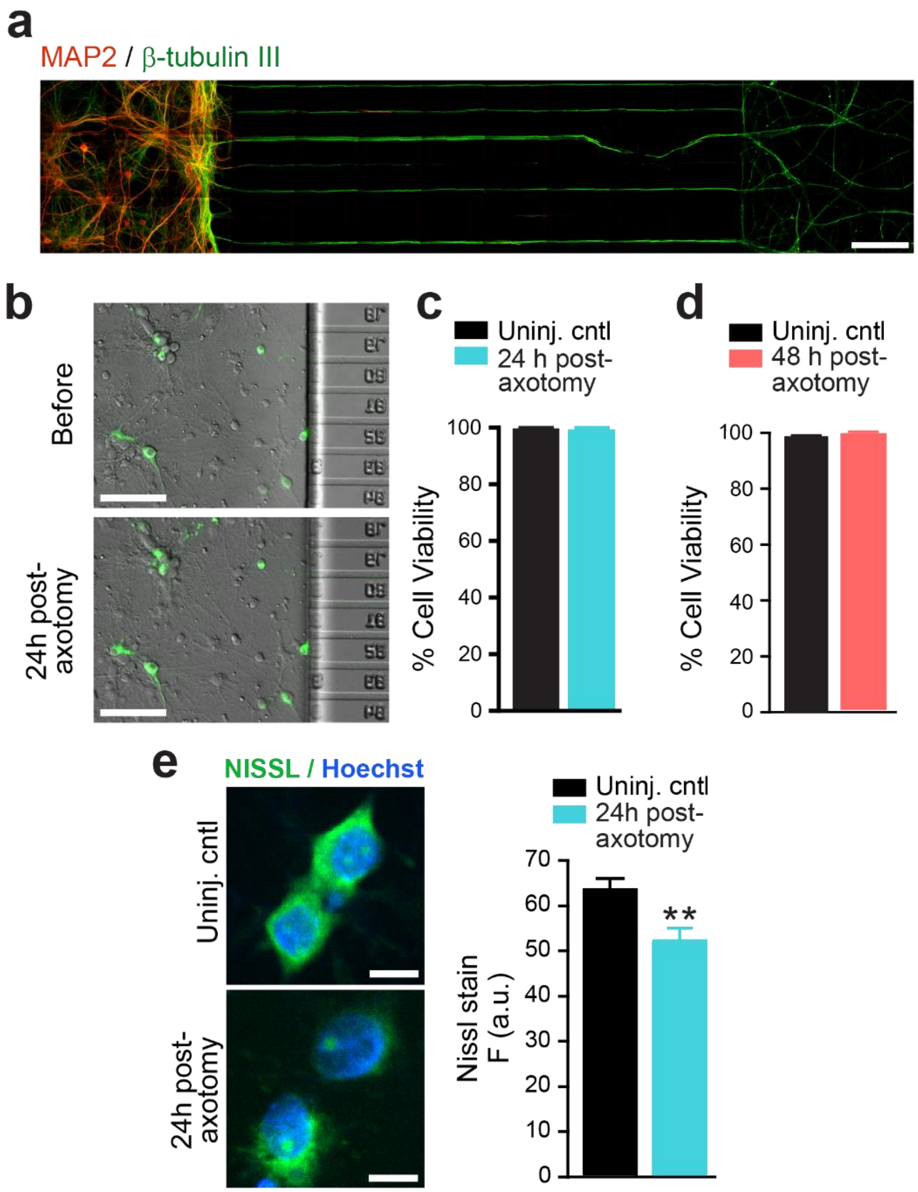

Supplementary Figure 1: Distal axotomy of pyramidal neurons induces dissolution of Nissl substance without affecting cell viability. (a) Images of cultured neurons within 900 µm barrier microfluidic chambers immunostained for the dendritic marker, MAP2, and neuron-specific, β-tubulin III. MAP2 staining is restricted to the somatodendritic compartment. The microfluidic chamber was removed during immunostaining to enable staining of axons and dendrites within the microgroove region. The removal of the chamber caused occasional shifting of axons as observed in this micrograph. (b)

Neurons, retrograde-labeled with an Alexa 488-conjugated cholera toxin, show no observable change in appearance following injury. Scale bar, 100 µm. (c) Cell viability at 24 h post-axotomy (control, n=830 neurons; axotomy, n=686 neurons). Results are from one of three experiments showing the same trend. (d) Cell viability at 48 h post-axotomy. *Control*: n= 560 neurons; 2 chambers per condition. *Axotomy*, n= 417 neurons; 2 chambers per condition. Results are from one of three experiments showing the same trend. (e) Nissl level decreases in the cell body of injured neurons identified using the retrograde tracer, Alexa 488-conjugated cholera toxin, at 24 h post-axotomy. *control*: n=19 neurons; 2 chambers per condition. *Axotomy*: n=18 neurons; 2 chambers per condition. Scale bar, 10 µm. Two-tailed t-test, ** $p < 0.01$. Error bars, s.e.m.

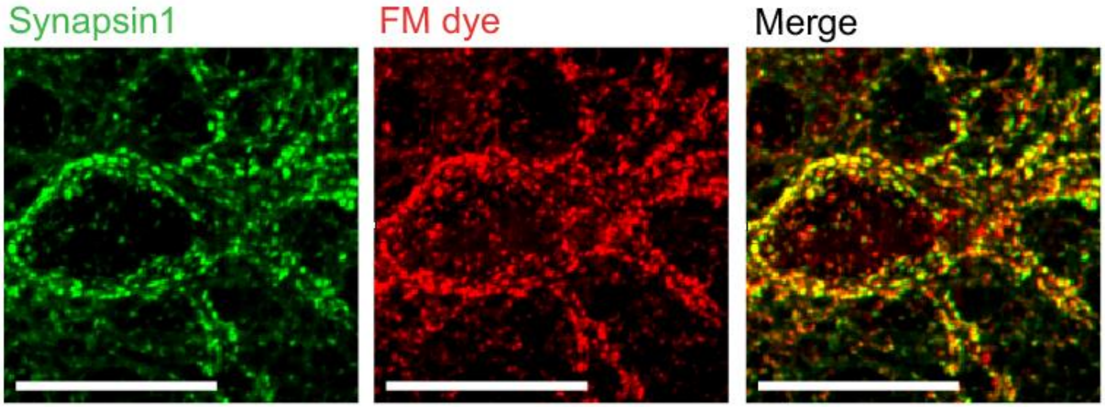

Supplementary Figure 2: FM puncta colocalize with synapsin1 immunolabeling. Representative images of neurons loaded with FM dye (FM4-64FX; red) into recycling synaptic vesicles and counterstained with synapsin1 antibody (green). Merge image indicate that FM labeled terminals are co-labeled with synapsin1. Pearson’s correlation coefficient, r=0.75. About 93% of FM labeled terminal expressed synapsin1 labeling. Results are from one of three experiments showing the same trend. Scale bars, 20 µm.

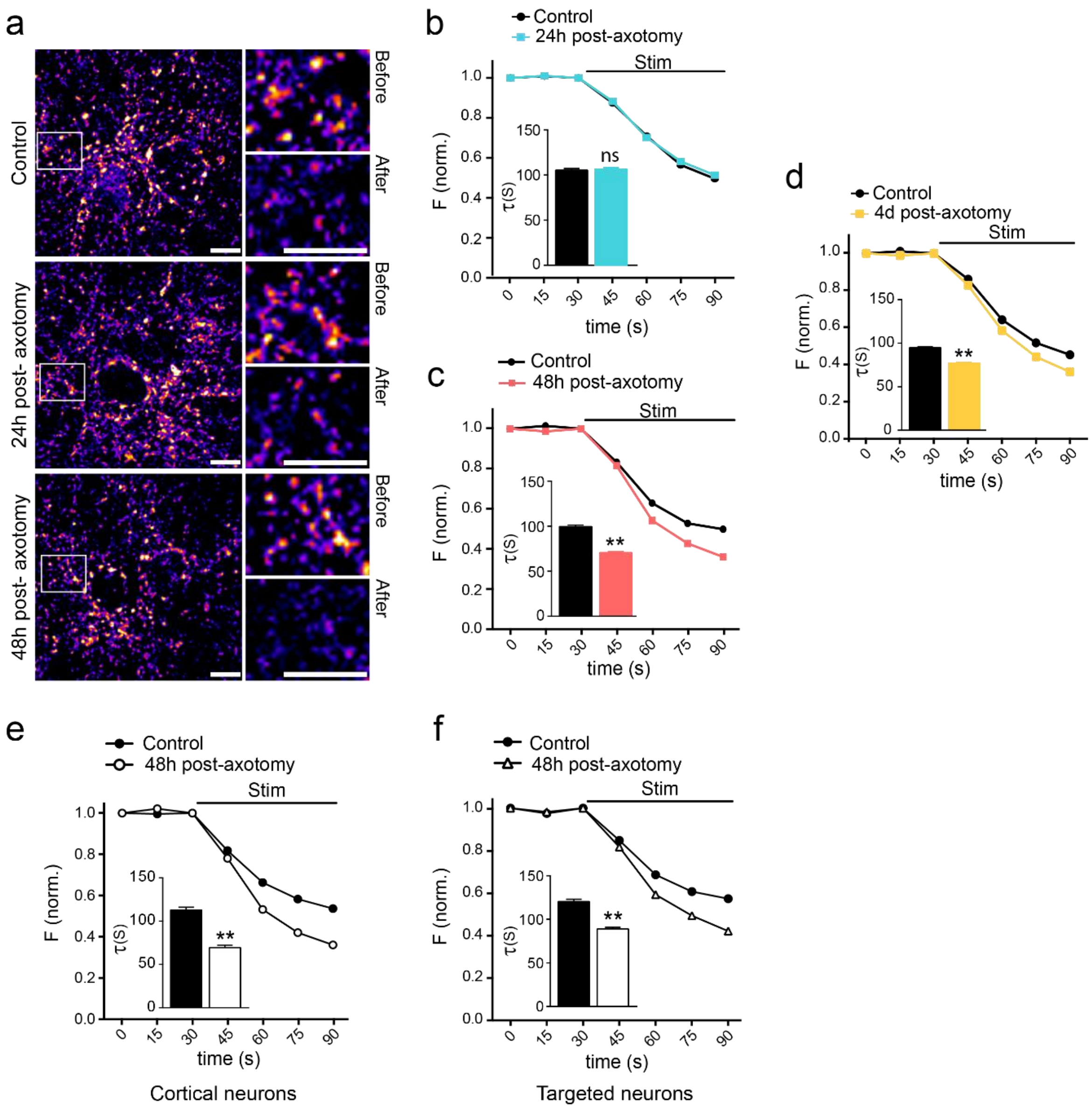

Supplementary Figure 3: FM unloading curves for (1) somatodendritic compartments, near the microgrooves of microfluidic chambers without retrograde labeling, and (2) cortical and targeted neuron populations subjected to axotomy. (a) Images within the somatodendritic compartment of microfluidic chambers showing presynaptic terminals labeled with FM5-95 (FM puncta). Color look up table 'Fire'. Enlarged outlined region shows FM puncta before and after field stimulation. Scale bars, 10 μm. (b) FM unloading curves 24 h post-axotomy (control, n=1,972 puncta; axotomy, n=1,944 puncta; 6 chambers for each condition). Inset shows mean time constant of unloading puncta. (c) 48 h

post-axotomy (control, n=2,368 puncta; axotomy, n=2,939 puncta; 6 chambers for each condition). (d) 4 d post-axotomy (control, n=3,438 puncta; axotomy, n=3,168 puncta; 6 chambers for each condition). (e) Cortical neurons show consistent FM unloading patterns as hippocampal neurons following axotomy (control, n=762 puncta; axotomy, n=482 puncta; 7 chambers for each condition). (f) Cultures with target neurons within the axonal compartment that are axotomized show consistent FM unloading patterns as axotomized untargeted axons (control, n=1,237 puncta; axotomy, n=1,602 puncta; 6 chambers for each condition). Data sets include 3 experiments. Error bars, s.e.m. **$p < 0.0001$.

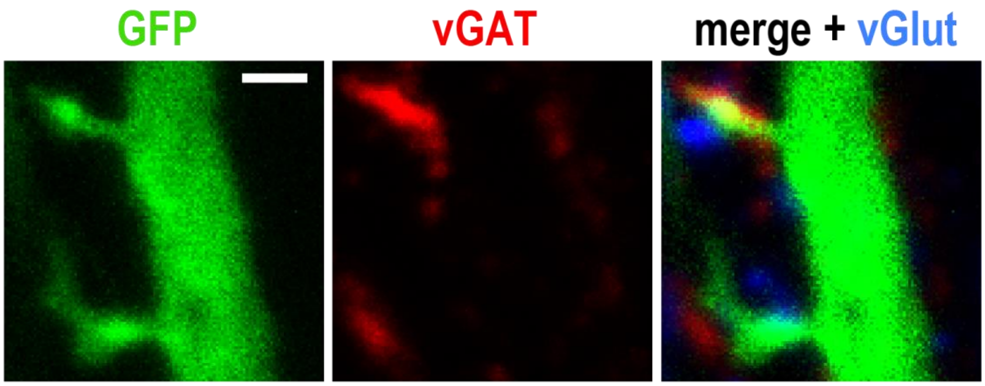

Supplementary Figure 4: Fluorescence micrograph of a dually innervated dendritic spine receiving both excitatory and inhibitory inputs. An example of a spine receiving dual innervation from both inhibitory (vGAT-positive, red) and excitatory (vGlut-positive, blue) presynaptic terminals. The GFP-filled dendritic segment was retrograde labeled within microfluidic chambers using a modified eGFP rabies virus. Roughly ~10% of spines are dually innervated (i.e., inhibited spines) in microfluidic cultures. Scale, 1 μm.

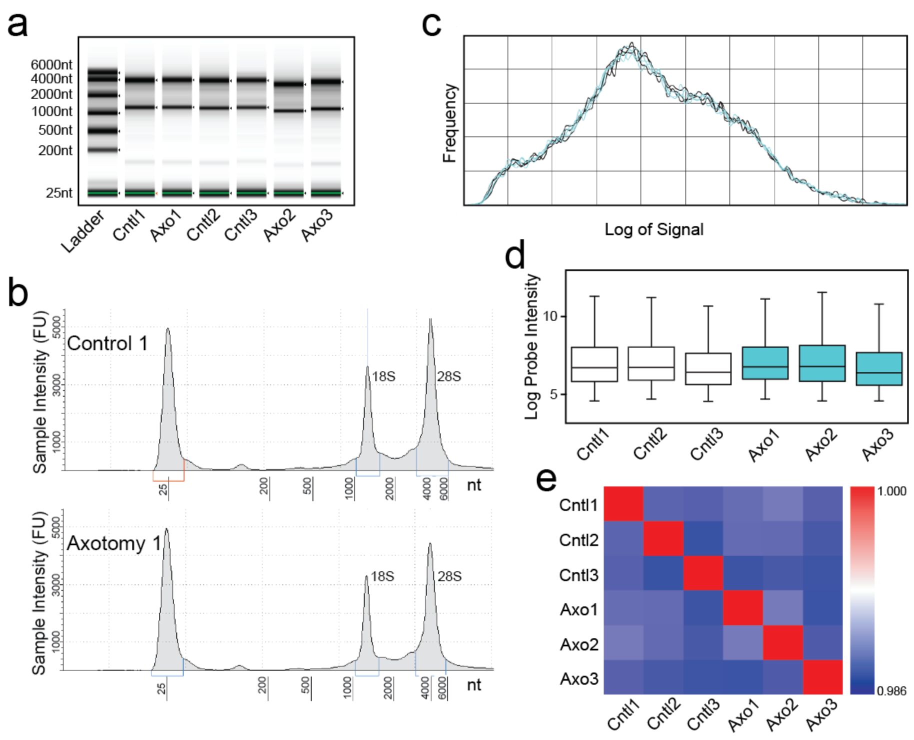

Supplementary Figure 5: RNA quality assessment and verification of microarray quality controls. (**a**) Image of ScreenTape gel from TapeStation RNA quality assessment of triplicate control (Cntl) and axotomy (Axo) RNA samples, standard ladder is included. (**b**) Fluorescence intensity traces of ScreenTape lanes from Control1 and Axotomy1 samples plotted versus nucleotide length as determined by the ladder. Clearly defined 18S and 28S bands in the gel (*a*) and peaks in the traces (*b*) indicate high quality RNA samples. (**c**) Histogram of microarray probe cell intensity following RMA analysis, plotted as frequency of signal versus signal intensity (log scale) for all control (black) and axotomy (blue) arrays. (**d**) Box-and-whiskers plot of the probe cell intensity (log scale) for each of the triplicate control (Cntl, white) and axotomy (Axo, blue) arrays following RMA analysis prior to normalization. (**e**) Pearson's correlation of normalized expression signal after analysis of triplicate control (Cntl) and axotomy (Axo) arrays. Indicator to the right demonstrates degree of correlation between samples.

Supplementary Table 1: Membrane properties of uninjured controls vs. axotomized neurons 48 h post-axotomy.

	Uninjured control (±SEM)	Axotomy (±SEM)	p-value
Sample Size (# neurons)	17	20	
C_m (pF)	51.9±4.5	57.0±4.6	0.44
R_s (MΩ)	13.1±0.8	13.2±1.0	0.90
R_m (MΩ)	715±108	661±92	0.39
Tau (μs)	281±33	331±52	0.77

Supplementary Table 2: List of transcripts that were significantly changed 24 h after injury.

Transcript Cluster ID	Injury Bi-weight Avg Signal (log2)	Control Bi-weight Avg Signal (log2)	Fold Change (linear) (Injury vs. Control)	Fold Change (log2)	ANOVA p-value (Injury vs. Control)	FDR p-value (Injury vs. Control)	Gene Symbol
17619431	3.75	2.72	2.03	1.0214797	0.028761	0.999562	Olr272
17610865	4.56	3.72	1.79	0.8399596	0.023334	0.999562	RGD1560695
17683217	3.97	3.14	1.77	0.8237494	0.001085	0.999562	RGD1560186; RGD1561310; RPL37-PS1
17802851	6.15	5.44	1.64	0.7136958	0.026	0.999562	LOC100359438
17667757	3.85	3.16	1.61	0.6870607	0.012066	0.999562	Gm6358
17839177	7.25	6.6	1.56	0.641546	0.044312	0.999562	Kdelr3
17826908	3.79	3.15	1.55	0.6322682	0.001538	0.999562	Vom1r106
17882823	7.39	6.83	1.48	0.5655972	0.021212	0.999562	LOC688318; Cyp27b1; Chchd2; LOC684871; Fabp5; Lce1l; Lsm2; LOC100362916; LOC682812; LOC684372; LOC100366231; LOC682352; LOC100910788; LOC501317; LOC684892; LOC100909462; LOC689635; LOC100909409; LOC100911002; LOC100910163; LOC100911516; RGD1561986; rno-mir-3584
17882811	11.07	10.53	1.45	0.5360529	0.003929	0.999562	LOC688318; Cyp27b1; Chchd2; LOC684871; Fabp5; Lce1l; Lsm2; LOC100362916; LOC682812; LOC684372; LOC100366231; LOC682352; LOC100910788; LOC501317; LOC684892; LOC100909462; LOC689635; LOC100909409; LOC100911002; LOC100910163; LOC100911516; RGD1561986; rno-mir-3584
17882813	11.07	10.53	1.45	0.5360529	0.003929	0.999562	LOC688318; Cyp27b1; Chchd2; LOC684871; Fabp5; Lce1l; Lsm2; LOC100362916; LOC682812; LOC684372; LOC100366231; LOC682352; LOC100910788; LOC501317; LOC684892; LOC100909462; LOC689635; LOC100909409; LOC100911002; LOC100910163; LOC100911516; RGD1561986; rno-mir-3584
17882815	11.07	10.53	1.45	0.5360529	0.003929	0.999562	LOC688318; Cyp27b1; Chchd2; LOC684871; Fabp5; Lce1l; Lsm2; LOC100362916; LOC682812; LOC684372; LOC100366231; LOC682352; LOC100910788; LOC501317; LOC684892; LOC100909462; LOC689635; LOC100909409; LOC100911002; LOC100910163; LOC100911516; RGD1561986; rno-mir-3584
17851313	4.09	3.55	1.45	0.5360529	0.010949	0.999562	Olr1303; LOC100910667
17786439	4.36	3.82	1.45	0.5360529	0.023165	0.999562	Olr824
17622003	3.03	2.51	1.43	0.5160151	0.032091	0.999562	Olr310
17728535	5.52	5.02	1.41	0.4956952	0.010546	0.999562	LOC683963
17808683	10.16	9.67	1.41	0.4956952	0.036579	0.999562	Jun
17789529	9.07	8.57	1.41	0.4956952	0.036746	0.999562	Bet1
17732587	3.17	2.67	1.41	0.4956952	0.03922	0.999562	Mir24-2; rno-mir-24-2
17638641	6.95	6.49	1.38	0.4646683	0.016001	0.999562	Ins2
17715387	4.56	4.09	1.38	0.4646683	0.036402	0.999562	Serpinb1a
17795789	3.99	3.54	1.37	0.4541759	0.009417	0.999562	LOC100360076
17856878	6.59	6.15	1.36	0.4436067	0.004841	0.999562	Slc5a7
17630022	6.71	6.27	1.36	0.4436067	0.012304	0.999562	Ehd2
17763726	4.07	3.62	1.36	0.4436067	0.041519	0.999562	Olr639; Olr636; LOC100910556; LOC100910858
17882299	4.63	4.2	1.35	0.4329594	0.012345	0.999562	LOC688318; Cyp27b1; Chchd2; LOC684871; Fabp5; Lce1l; Lsm2; LOC100362916; LOC682812; LOC684372; LOC100366231; LOC682352; LOC100910788; LOC501317; LOC684892; LOC100909462; LOC689635; LOC100909409; LOC100911002; LOC100910163; LOC100911516; RGD1561986; rno-mir-3584
17774130	3.06	2.63	1.35	0.4329594	0.025611	0.999562	Olr488
17774142	4.52	4.08	1.35	0.4329594	0.025612	0.999562	Olr499; LOC100912540
17759755	6.55	6.12	1.35	0.4329594	0.045664	0.999562	LOC679914
17651601	4.75	4.33	1.34	0.422233	0.013397	0.999562	Ccdc103
17834373	4.82	4.4	1.34	0.422233	0.01951	0.999562	RGD1560291
17881026	5.09	4.67	1.33	0.4114262	0.006697	0.999562	LOC685095
17621115	5.46	5.06	1.32	0.4005379	0.012195	0.999562	Itgax
17782183	5.27	4.87	1.32	0.4005379	0.017396	0.999562	Ssmem1
17658475	4.48	4.08	1.32	0.4005379	0.040871	0.999562	Olr1470; Olr1481; LOC100909493
17658462	5.91	5.52	1.31	0.3895668	0.033909	0.999562	Aspa

Transcript Cluster ID	Injury Bi-weight Avg Signal (log2)	Control Bi-weight Avg Signal (log2)	Fold Change (linear) (Injury vs. Control)	Fold Change (log2)	ANOVA p-value (Injury vs. Control)	FDR p-value (Injury vs. Control)	Gene Symbol
17880923	2.96	2.57	1.31	0.3895668	0.04276	0.999562	Olr1291; LOC100911015; LOC100911064
17777579	5.75	5.37	1.3	0.3785116	0.007331	0.999562	Lrrn4
17612506	5.99	5.62	1.3	0.3785116	0.046768	0.999562	Zfp52
17656475	5.11	4.74	1.29	0.3673711	0.021102	0.999562	Olr1423
17851236	4.18	3.81	1.29	0.3673711	0.021408	0.999562	Olr1273; LOC100909937; LOC100909958; LOC100910086; LOC100910153; LOC100910227; LOC100910363; LOC100910548
17666651	5.05	4.69	1.28	0.3561438	0.01255	0.999562	Crygs
17882371	4.92	4.57	1.28	0.3561438	0.040726	0.999562	LOC688318; Cyp27b1; Chchd2; LOC684871; Fabp5; Lce1l; Lsm2; LOC100362916; LOC682812; LOC684372; LOC100366231; LOC682352; LOC100910788; LOC501317; LOC684892; LOC100909462; LOC689635; LOC100909409; LOC100911002; LOC100910163; LOC100911516; RGD1561986; rno-mir-3584
17843379	4.72	4.37	1.28	0.3561438	0.049296	0.999562	Olr1308
17851371	3.68	3.32	1.28	0.3561438	0.049325	0.999562	Olr1338
17833268	5.34	4.99	1.27	0.3448285	0.038378	0.999562	Olr1061
17765578	4.62	4.28	1.27	0.3448285	0.047367	0.999562	Duoxa2
17783058	4.99	4.65	1.26	0.3334237	0.0119	0.999562	Tas2r143; LOC100911989
17769970	8.27	7.94	1.26	0.3334237	0.025078	0.999562	Spopl
17731468	6.72	6.38	1.26	0.3334237	0.049326	0.999562	Tepp
17751632	4.93	4.61	1.25	0.3219281	0.000356	0.876358	RGD1309170
17752004	6.48	6.16	1.25	0.3219281	0.0021	0.999562	Ifi44
17824773	3.34	3.01	1.25	0.3219281	0.003067	0.999562	LOC100912547; LOC100911906
17692803	4.74	4.42	1.25	0.3219281	0.01544	0.999562	Abcg3l1
17850026	3	2.67	1.25	0.3219281	0.017621	0.999562	Olr1171
17698136	6.3	5.98	1.25	0.3219281	0.029601	0.999562	Rnase1l2
17790903	4.77	4.45	1.24	0.3103401	0.006892	0.999562	Clec5a
17881701	7.3	6.98	1.24	0.3103401	0.017742	0.999562	LOC688318; Cyp27b1; Chchd2; LOC684871; Fabp5; Lce1l; Lsm2; LOC100362916; LOC682812; LOC684372; LOC100366231; LOC682352; LOC100910788; LOC501317; LOC684892; LOC100909462; LOC689635; LOC100909409; LOC100911002; LOC100910163; LOC100911516; RGD1561986; rno-mir-3584
17782972	2.6	2.3	1.24	0.3103401	0.03498	0.999562	Sval1
17741173	10.23	9.93	1.24	0.3103401	0.047338	0.999562	Sec22b
17629376	3.6	3.29	1.23	0.2986583	0.002959	0.999562	Olr7
17613026	6.66	6.36	1.23	0.2986583	0.011027	0.999562	Isoc2b
17800945	4.15	3.86	1.23	0.2986583	0.033823	0.999562	Agbl4
17719288	3.12	2.83	1.23	0.2986583	0.038188	0.999562	Prl3a1
17686320	4.01	3.71	1.23	0.2986583	0.039704	0.999562	LOC685351
17797676	6.12	5.82	1.23	0.2986583	0.045257	0.999562	RGD1562265
17864319	5.7	5.41	1.22	0.2868811	0.005712	0.999562	Mstn
17827861	8.45	8.17	1.22	0.2868811	0.032067	0.999562	Kitlg
17881843	4.11	3.83	1.22	0.2868811	0.038699	0.999562	LOC688318; Cyp27b1; Chchd2; LOC684871; Fabp5; Lce1l; Lsm2; LOC100362916; LOC682812; LOC684372; LOC100366231; LOC682352; LOC100910788; LOC501317; LOC684892; LOC100909462; LOC689635; LOC100909409; LOC100911002; LOC100910163; LOC100911516; RGD1561986; rno-mir-3584
17790891	4.83	4.55	1.22	0.2868811	0.03951	0.999562	Prss37
17868867	5.89	5.6	1.22	0.2868811	0.041644	0.999562	LOC291863
17879514	6.4	6.11	1.22	0.2868811	0.042099	0.999562	Mcf2
17876558	6.38	6.1	1.22	0.2868811	0.048286	0.999562	Zcchc16
17730018	5.2	4.92	1.21	0.275007	0.010621	0.999562	Tat
17685608	8.41	8.14	1.21	0.275007	0.012676	0.999562	Soat1
17842112	6.61	6.33	1.21	0.275007	0.038701	0.999562	Vstm5
17746221	7.25	6.97	1.21	0.275007	0.040677	0.999562	Car3

Transcript Cluster ID	Injury Bi-weight Avg Signal (log2)	Control Bi-weight Avg Signal (log2)	Fold Change (linear) (Injury vs. Control)	Fold Change (log2)	ANOVA p-value (Injury vs. Control)	FDR p-value (Injury vs. Control)	Gene Symbol
17881561	4	3.73	1.2	0.2630344	0.004532	0.999562	LOC688318; Cyp27b1; Chchd2; LOC684871; Fabp5; Lce1l; Lsm2; LOC100362916; LOC682812; LOC684372; LOC100366231; LOC682352; LOC100910788; LOC501317; LOC684892; LOC100909462; LOC689635; LOC100909409; LOC100911002; LOC100910163; LOC100911516; RGD1561986; rno-mir-3584
17744282	8.34	8.08	1.2	0.2630344	0.005696	0.999562	Papd4
17822715	4.98	4.72	1.2	0.2630344	0.006277	0.999562	Tex21
17762362	5.53	5.26	1.2	0.2630344	0.013101	0.999562	LOC100361645
17763581	3.81	3.54	1.2	0.2630344	0.013667	0.999562	Olr456
17779084	9.17	8.9	1.2	0.2630344	0.019641	0.999562	Oser1
17629411	8.24	7.98	1.2	0.2630344	0.02397	0.999562	Zfp667
17769590	9.36	9.1	1.2	0.2630344	0.028284	0.999562	Ogfr
17695584	5.58	5.32	1.2	0.2630344	0.034689	0.999562	Nkx1-1
17612006	9.34	9.07	1.2	0.2630344	0.036269	0.999562	Rnaset2
17626755	5.78	5.52	1.2	0.2630344	0.041758	0.999562	Pnlip
17615156	6.91	6.65	1.2	0.2630344	0.042974	0.999562	Zfp790; LOC100359464
17812266	5.49	5.22	1.2	0.2630344	0.043536	0.999562	RGD1306186
17881080	9.85	9.6	1.19	0.2509616	0.011961	0.999562	LOC687711
17723983	5.86	5.61	1.19	0.2509616	0.026427	0.999562	Cidea
17877518	6.46	6.21	1.19	0.2509616	0.031518	0.999562	Prrg1
17635086	6.79	6.54	1.19	0.2509616	0.037607	0.999562	Acer3
17646510	8.74	8.5	1.18	0.2387869	0.003092	0.999562	Alkbh5
17734361	6.77	6.53	1.18	0.2387869	0.004793	0.999562	Acta1
17835237	7.06	6.82	1.18	0.2387869	0.0136	0.999562	Elk3
17699671	6.19	5.95	1.18	0.2387869	0.016376	0.999562	Scara5
17792908	9.97	9.73	1.18	0.2387869	0.030267	0.999562	Rpl22
17833304	8.18	7.94	1.18	0.2387869	0.031989	0.999562	Zfp709l1
17705822	4.99	4.75	1.18	0.2387869	0.032546	0.999562	Hesx1
17788445	6.28	6.05	1.18	0.2387869	0.034422	0.999562	Htr5a
17825419	7.6	7.36	1.18	0.2387869	0.038025	0.999562	Cwf19l2
17830560	7.08	6.84	1.18	0.2387869	0.041384	0.999562	Micall1
17881101	4.68	4.45	1.17	0.2265085	0.00648	0.999562	Or51t1
17745409	6.28	6.06	1.17	0.2265085	0.006963	0.999562	Isl1
17685125	7.28	7.05	1.17	0.2265085	0.011292	0.999562	Swt1
17619191	3.56	3.34	1.17	0.2265085	0.027472	0.999562	LOC689730
17752139	6.67	6.45	1.17	0.2265085	0.030748	0.999562	Tyw3
17672845	8.79	8.56	1.17	0.2265085	0.034368	0.999562	Prkrip1
17615690	4.62	4.39	1.17	0.2265085	0.036049	0.999562	LOC690284
17830057	10.4	10.17	1.17	0.2265085	0.036966	0.999562	Grina
17807952	6.65	6.42	1.17	0.2265085	0.049929	0.999562	Hdhd3
17612996	5.76	5.54	1.16	0.2141248	0.000694	0.999562	Rfpl4a
17812245	9.98	9.76	1.16	0.2141248	0.004167	0.999562	Pdpn
17811191	5.96	5.74	1.16	0.2141248	0.006542	0.999562	Fgr
17873035	4.74	4.53	1.16	0.2141248	0.01315	0.999562	Awat1
17862568	7.09	6.88	1.16	0.2141248	0.014239	0.999562	Trem2
17881551	3.4	3.19	1.16	0.2141248	0.025286	0.999562	LOC688318; Cyp27b1; Chchd2; LOC684871; Fabp5; Lce1l; Lsm2; LOC100362916; LOC682812; LOC684372; LOC100366231; LOC682352; LOC100910788; LOC501317; LOC684892; LOC100909462; LOC689635; LOC100909409; LOC100911002; LOC100910163; LOC100911516; RGD1561986; rno-mir-3584
17873568	4.11	3.89	1.16	0.2141248	0.025841	0.999562	RGD1562871
17669931	7.38	7.17	1.16	0.2141248	0.026271	0.999562	Fam43a
17641633	4.58	4.37	1.16	0.2141248	0.034034	0.999562	RGD1565682
17757261	5.85	5.64	1.16	0.2141248	0.039772	0.999562	LOC100360640
17766543	9.52	9.3	1.16	0.2141248	0.040156	0.999562	Crls1
17800346	6.25	6.03	1.16	0.2141248	0.04244	0.999562	Oma1
17856971	6.61	6.4	1.16	0.2141248	0.049934	0.999562	RGD1559808
17622676	8.69	8.48	1.15	0.2016339	0.012715	0.999562	Cpt1a

Transcript Cluster ID	Injury Bi-weight Avg Signal (log2)	Control Bi-weight Avg Signal (log2)	Fold Change (linear) (Injury vs. Control)	Fold Change (log2)	ANOVA p-value (Injury vs. Control)	FDR p-value (Injury vs. Control)	Gene Symbol
17881623	8.91	8.71	1.15	0.2016339	0.013024	0.999562	LOC688318; Cyp27b1; Chchd2; LOC684871; Fabp5; Lce1l; Lsm2; LOC100362916; LOC682812; LOC684372; LOC100366231; LOC682352; LOC100910788; LOC501317; LOC684892; LOC100909462; LOC689635; LOC100909409; LOC100911002; LOC100910163; LOC100911516; RGD1561986; rno-mir-3584
17719453	5.63	5.43	1.15	0.2016339	0.020199	0.999562	Cmah
17852165	6.12	5.92	1.15	0.2016339	0.020203	0.999562	Htr3a
17823379	6.59	6.38	1.15	0.2016339	0.023366	0.999562	Tgfb3
17812591	9.59	9.38	1.15	0.2016339	0.036955	0.999562	Draxin
17822377	5.73	5.53	1.15	0.2016339	0.048562	0.999562	Cdkl1
17651411	6.37	6.18	1.14	0.1890338	0.000454	0.897373	Rdm1
17735006	8.06	7.87	1.14	0.1890338	0.002392	0.999562	Lysmd3
17859309	7.63	7.44	1.14	0.1890338	0.005365	0.999562	MGC94335
17859543	6.22	6.03	1.14	0.1890338	0.022635	0.999562	Casp8
17868542	6.83	6.64	1.14	0.1890338	0.02922	0.999562	Steap3
17619435	4.03	3.84	1.14	0.1890338	0.031578	0.999562	Olr276
17770946	7.34	7.15	1.14	0.1890338	0.034925	0.999562	RGD1306233
17740203	9.37	9.19	1.14	0.1890338	0.039086	0.999562	LOC361985
17817302	7.49	7.3	1.14	0.1890338	0.043596	0.999562	Acot2
17816111	7.57	7.38	1.14	0.1890338	0.045216	0.999562	RGD1307621
17780046	5.09	4.9	1.14	0.1890338	0.046364	0.999562	Bhlhe23
17735614	8.46	8.27	1.14	0.1890338	0.048846	0.999562	Dimt1
17656730	8.59	8.41	1.13	0.1763228	0.002642	0.999562	Flcn
17623100	8.26	8.07	1.13	0.1763228	0.007188	0.999562	Eif1ad
17710214	5.4	5.23	1.13	0.1763228	0.013499	0.999562	Slc18a3
17765048	5.69	5.52	1.13	0.1763228	0.013634	0.999562	Spint1
17680143	9	8.83	1.13	0.1763228	0.016847	0.999562	Ppp1r15b
17679753	5.44	5.26	1.13	0.1763228	0.018811	0.999562	Map3k19
17865147	9.02	8.84	1.13	0.1763228	0.02085	0.999562	Acadl
17882105	6.43	6.26	1.13	0.1763228	0.028186	0.999562	LOC688318; Cyp27b1; Chchd2; LOC684871; Fabp5; Lce1l; Lsm2; LOC100362916; LOC682812; LOC684372; LOC100366231; LOC682352; LOC100910788; LOC501317; LOC684892; LOC100909462; LOC689635; LOC100909409; LOC100911002; LOC100910163; LOC100911516; RGD1561986; rno-mir-3584
17714388	6.2	6.03	1.13	0.1763228	0.031397	0.999562	Dok3
17766437	7.38	7.21	1.13	0.1763228	0.035882	0.999562	Mavs
17880424	9.62	9.44	1.13	0.1763228	0.035967	0.999562	Dmtf1; LOC681224
17649180	6.95	6.77	1.13	0.1763228	0.037411	0.999562	Mir423; rno-mir-423
17722342	8.99	8.82	1.13	0.1763228	0.038532	0.999562	Fam53c
17792516	6.76	6.59	1.13	0.1763228	0.046155	0.999562	Hk2
17870547	5.85	5.67	1.13	0.1763228	0.049267	0.999562	Rhox2
17650204	8.19	8.03	1.12	0.1634987	0.000706	0.999562	Cox11
17669356	9.33	9.16	1.12	0.1634987	0.001248	0.999562	Kpna1
17823218	10.45	10.29	1.12	0.1634987	0.00742	0.999562	Npc2
17749936	7.93	7.76	1.12	0.1634987	0.008305	0.999562	Ptgfrn
17870502	7.8	7.64	1.12	0.1634987	0.013967	0.999562	Tmem255a
17860426	8.27	8.11	1.12	0.1634987	0.018721	0.999562	Plcd4
17851854	10.39	10.23	1.12	0.1634987	0.021625	0.999562	Arcn1
17616249	7.3	7.13	1.12	0.1634987	0.028126	0.999562	Tead2
17658885	5.77	5.6	1.12	0.1634987	0.028698	0.999562	Proca1
17616399	7.77	7.61	1.12	0.1634987	0.028777	0.999562	Bcat2
17847108	6.92	6.76	1.12	0.1634987	0.029366	0.999562	Acad11
17717629	9.7	9.54	1.12	0.1634987	0.029827	0.999562	Nsun2
17817610	5.83	5.66	1.12	0.1634987	0.031412	0.999562	Esrrb
17673751	5.58	5.42	1.12	0.1634987	0.032506	0.999562	Gpr81
17873000	6.88	6.71	1.12	0.1634987	0.03444	0.999562	Fam155b
17635008	8.05	7.89	1.12	0.1634987	0.038878	0.999562	Aqp11
17783073	5.02	4.85	1.12	0.1634987	0.040603	0.999562	Olr809

Transcript Cluster ID	Injury Bi-weight Avg Signal (log2)	Control Bi-weight Avg Signal (log2)	Fold Change (linear) (Injury vs. Control)	Fold Change (log2)	ANOVA p-value (Injury vs. Control)	FDR p-value (Injury vs. Control)	Gene Symbol
17882091	7.05	6.89	1.12	0.1634987	0.044003	0.999562	LOC688318; Cyp27b1; Chchd2; LOC684871; Fabp5; Lce1l; Lsm2; LOC100362916; LOC682812; LOC684372; LOC100366231; LOC682352; LOC100910788; LOC501317; LOC684892; LOC100909462; LOC689635; LOC100909409; LOC100911002; LOC100910163; LOC100911516; RGD1561986; rno-mir-3584
17805564	8.95	8.79	1.12	0.1634987	0.047511	0.999562	Tcea1; LOC498453
17872966	9.37	9.2	1.12	0.1634987	0.047722	0.999562	Yipf6
17718585	8.78	8.63	1.11	0.1505597	0.000137	0.832906	LOC100911018
17876823	9.25	9.1	1.11	0.1505597	0.00031	0.876358	Tsr2
17636538	10.9	10.74	1.11	0.1505597	0.001665	0.999562	Rps15a; LOC691065
17678672	9.26	9.11	1.11	0.1505597	0.002356	0.999562	Triap1; LOC100910137
17785409	6	5.85	1.11	0.1505597	0.002698	0.999562	LOC685964
17775350	5.37	5.22	1.11	0.1505597	0.007774	0.999562	Chrm5
17864008	9.78	9.64	1.11	0.1505597	0.015782	0.999562	Txndc9
17809602	8.82	8.67	1.11	0.1505597	0.030173	0.999562	Urod
17821879	7.69	7.54	1.11	0.1505597	0.033215	0.999562	Egln3
17716240	10.66	10.51	1.11	0.1505597	0.035005	0.999562	Rab18
17695236	6.54	6.38	1.11	0.1505597	0.036972	0.999562	Hmx1
17618226	5.9	5.75	1.11	0.1505597	0.038931	0.999562	Ccdc89
17785641	10.74	10.59	1.11	0.1505597	0.04035	0.999562	Arl6ip5
17878832	9.33	9.18	1.11	0.1505597	0.041242	0.999562	Armcx2
17859930	8.63	8.48	1.11	0.1505597	0.042076	0.999562	Creb1
17648712	7.35	7.2	1.11	0.1505597	0.043874	0.999562	RGD1309077
17834867	9.42	9.28	1.11	0.1505597	0.044037	0.999562	Chpt1
17769017	6.33	6.19	1.11	0.1505597	0.048053	0.999562	Slc2a10
17611290	9.7	9.55	1.11	0.1505597	0.048636	0.999562	Pdcd6
17835359	9.9	9.76	1.11	0.1505597	0.049307	0.999562	Nudt4
17861494	7.37	7.23	1.1	0.1375035	0.001137	0.999562	Cxcr7
17818342	8.99	8.85	1.1	0.1375035	0.002856	0.999562	Yy1
17837024	7.63	7.49	1.1	0.1375035	0.007097	0.999562	Slc25a32
17782065	10.1	9.97	1.1	0.1375035	0.007237	0.999562	Atp6v1f
17668877	9.34	9.2	1.1	0.1375035	0.008511	0.999562	Atg3
17651309	7.66	7.52	1.1	0.1375035	0.011046	0.999562	G6pc; Aoc3; Psme3
17750404	8.68	8.54	1.1	0.1375035	0.015557	0.999562	Gstm7
17627328	7.43	7.28	1.1	0.1375035	0.016264	0.999562	Ltv1
17658044	9.15	9.01	1.1	0.1375035	0.016396	0.999562	RGD1308134
17758309	9.93	9.8	1.1	0.1375035	0.019305	0.999562	Ddit4
17684016	6.53	6.39	1.1	0.1375035	0.020544	0.999562	Il19
17777521	9.89	9.76	1.1	0.1375035	0.026123	0.999562	Tmem230
17711288	8.16	8.02	1.1	0.1375035	0.02974	0.999562	Tmem192
17881163	8.12	7.98	1.1	0.1375035	0.035015	0.999562	LOC100363176
17749347	7.99	7.85	1.1	0.1375035	0.036511	0.999562	LOC100363361
17881957	8.3	8.16	1.1	0.1375035	0.039617	0.999562	LOC688318; Cyp27b1; Chchd2; LOC684871; Fabp5; Lce1l; Lsm2; LOC100362916; LOC682812; LOC684372; LOC100366231; LOC682352; LOC100910788; LOC501317; LOC684892; LOC100909462; LOC689635; LOC100909409; LOC100911002; LOC100910163; LOC100911516; RGD1561986; rno-mir-3584
17878378	8.31	8.17	1.1	0.1375035	0.040649	0.999562	Magt1
17717908	5.21	5.08	1.1	0.1375035	0.041862	0.999562	Spata31d1b
17859846	11.32	11.19	1.1	0.1375035	0.042115	0.999562	Rpl37; LOC690840; LOC100360781; LOC100360841; LOC100365214
17757508	9.4	9.26	1.1	0.1375035	0.047452	0.999562	Cstb
17880368	12.05	11.91	1.1	0.1375035	0.047802	0.999562	LOC685718
17821215	8.59	8.47	1.09	0.1243281	0.005929	0.999562	Itgb1bp1
17803953	5.79	5.66	1.09	0.1243281	0.006438	0.999562	Agmat
17808953	4.11	3.99	1.09	0.1243281	0.007283	0.999562	RGD1562532
17751413	7.94	7.81	1.09	0.1243281	0.009696	0.999562	Ostc
17622272	10.84	10.71	1.09	0.1243281	0.012019	0.999562	Rplp2; LOC100911575
17834069	6.2	6.07	1.09	0.1243281	0.014101	0.999562	Giot1
17630055	6.28	6.15	1.09	0.1243281	0.014991	0.999562	Npas1
17816314	10.39	10.27	1.09	0.1243281	0.018597	0.999562	Tmx1
17786877	8.7	8.58	1.09	0.1243281	0.021103	0.999562	M6pr; LOC100909548

Transcript Cluster ID	Injury Bi-weight Avg Signal (log2)	Control Bi-weight Avg Signal (log2)	Fold Change (linear) (Injury vs. Control)	Fold Change (log2)	ANOVA p-value (Injury vs. Control)	FDR p-value (Injury vs. Control)	Gene Symbol
17773787	7.6	7.48	1.09	0.1243281	0.02919	0.999562	Zfp385b
17862257	8.28	8.16	1.09	0.1243281	0.02955	0.999562	Zfp161
17697062	5.35	5.22	1.09	0.1243281	0.03148	0.999562	Synpo2l
17854612	7.17	7.04	1.09	0.1243281	0.04352	0.999562	Tmed3
17870827	8.02	7.89	1.09	0.1243281	0.044503	0.999562	Ndp; LOC100909913
17821652	11.04	10.93	1.08	0.1110313	0.000793	0.999562	St13; LOC100362999
17854761	9.51	9.41	1.08	0.1110313	0.000832	0.999562	Atp1b3
17622041	5.53	5.42	1.08	0.1110313	0.001465	0.999562	Scgb1c1
17650636	8.7	8.59	1.08	0.1110313	0.00225	0.999562	Mrpl45
17843868	8.65	8.54	1.08	0.1110313	0.011693	0.999562	Zfp259
17675923	8.8	8.69	1.08	0.1110313	0.012996	0.999562	Bri3
17721797	6.41	6.31	1.08	0.1110313	0.014041	0.999562	Rnf125
17849209	9.38	9.27	1.08	0.1110313	0.014467	0.999562	Sacm1l
17694730	9.36	9.26	1.08	0.1110313	0.014958	0.999562	Anapc4
17653004	9.28	9.16	1.08	0.1110313	0.016542	0.999562	Sec14l1
17742074	6.91	6.79	1.08	0.1110313	0.016556	0.999562	Col11a1
17650526	6.04	5.93	1.08	0.1110313	0.016653	0.999562	Hoxb6
17669086	7.73	7.63	1.08	0.1110313	0.017546	0.999562	Tmem39a
17710636	9.66	9.55	1.08	0.1110313	0.022465	0.999562	Rab8a
17797345	7.72	7.61	1.08	0.1110313	0.02379	0.999562	Triqk
17861201	6.47	6.36	1.08	0.1110313	0.024538	0.999562	Efhd1
17804598	11.29	11.19	1.08	0.1110313	0.02481	0.999562	Rpl22
17726177	9.52	9.41	1.08	0.1110313	0.026158	0.999562	Atg12
17846248	5.65	5.54	1.08	0.1110313	0.026845	0.999562	Mei4
17705450	7.87	7.76	1.08	0.1110313	0.028316	0.999562	Tmtc4
17793715	9.56	9.45	1.08	0.1110313	0.029435	0.999562	Crbn
17821373	5.92	5.81	1.08	0.1110313	0.029863	0.999562	Pxdn
17717060	9.12	9.01	1.08	0.1110313	0.030804	0.999562	Prpf18
17785146	8.44	8.34	1.08	0.1110313	0.035476	0.999562	Fam136a
17827299	7.17	7.06	1.08	0.1110313	0.040085	0.999562	LOC691921
17826085	5.12	5.01	1.08	0.1110313	0.042384	0.999562	Gipc3
17849064	8.06	7.95	1.08	0.1110313	0.043149	0.999562	Tcaim
17783840	8.8	8.69	1.08	0.1110313	0.044391	0.999562	Avl9
17651928	7.32	7.21	1.08	0.1110313	0.044534	0.999562	Mrc2
17671438	8.93	8.83	1.08	0.1110313	0.044755	0.999562	N4bp2l2
17612316	8.73	8.62	1.08	0.1110313	0.045666	0.999562	Tbp
17823417	8.05	7.94	1.08	0.1110313	0.046015	0.999562	Pomt2
17733202	7.8	7.69	1.08	0.1110313	0.046237	0.999562	Psmb10
17624053	10.51	10.39	1.08	0.1110313	0.048529	0.999562	Ddb1
17654541	8	7.9	1.08	0.1110313	0.049172	0.999562	Tbl3
17652887	8.23	8.12	1.08	0.1110313	0.049566	0.999562	Unk
17715167	8.95	8.85	1.07	0.0976108	0.000491	0.897373	Eef1e1
17646169	4.01	3.92	1.07	0.0976108	0.006734	0.999562	LOC24906
17773252	6.95	6.85	1.07	0.0976108	0.010013	0.999562	Wipf1
17729695	9.94	9.84	1.07	0.0976108	0.011856	0.999562	LOC100360573
17730529	6.11	6.02	1.07	0.0976108	0.014693	0.999562	Osgin1
17736736	8.23	8.14	1.07	0.0976108	0.014993	0.999562	Myo10
17657793	8.47	8.36	1.07	0.0976108	0.015384	0.999562	Senp3
17709601	9.95	9.85	1.07	0.0976108	0.01653	0.999562	Appl1
17810783	9.02	8.92	1.07	0.0976108	0.02798	0.999562	Rbbp4
17778986	8.82	8.73	1.07	0.0976108	0.028727	0.999562	Zhx3
17666512	10.1	10	1.07	0.0976108	0.033503	0.999562	Fgf12
17733650	6.27	6.18	1.07	0.0976108	0.034956	0.999562	Fa2h
17797143	11.26	11.16	1.07	0.0976108	0.036244	0.999562	Sdcbp
17804980	10.15	10.04	1.07	0.0976108	0.038193	0.999562	LOC100364191
17882135	9.15	9.06	1.07	0.0976108	0.041024	0.999562	LOC688318; Cyp27b1; Chchd2; LOC684871; Fabp5; Lce1l; Lsm2; LOC100362916; LOC682812; LOC684372; LOC100366231; LOC682352; LOC100910788; LOC501317; LOC684892; LOC100909462; LOC689635; LOC100909409; LOC100911002; LOC100910163; LOC100911516; RGD1561986; rno-mir-3584
17753973	6.44	6.35	1.07	0.0976108	0.042163	0.999562	Slc37a1
17664499	5.95	5.85	1.07	0.0976108	0.044054	0.999562	LOC100363184
17715873	8.33	8.23	1.07	0.0976108	0.048985	0.999562	Zkscan3
17806052	3.02	2.94	1.06	0.0840643	0.001112	0.999562	Cnbd1

Transcript Cluster ID	Injury Bi-weight Avg Signal (log2)	Control Bi-weight Avg Signal (log2)	Fold Change (linear) (Injury vs. Control)	Fold Change (log2)	ANOVA p-value (Injury vs. Control)	FDR p-value (Injury vs. Control)	Gene Symbol
17656485	4.31	4.23	1.06	0.0840643	0.002418	0.999562	LOC686792
17848586	8.66	8.58	1.06	0.0840643	0.017032	0.999562	Abhd5
17826234	7.58	7.49	1.06	0.0840643	0.022432	0.999562	Plekhj1
17756721	4.63	4.55	1.06	0.0840643	0.028773	0.999562	Tesb
17619304	8.93	8.84	1.06	0.0840643	0.029134	0.999562	Ilk
17717253	8.53	8.45	1.06	0.0840643	0.036381	0.999562	Arl5b
17672137	7.31	7.22	1.06	0.0840643	0.036592	0.999562	Psmg3
17858306	7.71	7.63	1.06	0.0840643	0.043379	0.999562	Amer3
17881078	12.18	12.09	1.06	0.0840643	0.044446	0.999562	LOC100360791
17870139	8.94	8.86	1.06	0.0840643	0.045253	0.999562	Timmdc1
17787279	9.35	9.26	1.06	0.0840643	0.04684	0.999562	Mrpl51
17797006	8.35	8.28	1.05	0.0703893	0.017832	0.999562	Rgs20
17813740	7.19	7.12	1.05	0.0703893	0.023635	0.999562	Dync2li1
17667043	8.32	8.25	1.05	0.0703893	0.027932	0.999562	Prodh
17882015	7.75	7.68	1.05	0.0703893	0.03257	0.999562	LOC688318; Cyp27b1; Chchd2; LOC684871; Fabp5; Lce1l; Lsm2; LOC100362916; LOC682812; LOC684372; LOC100366231; LOC682352; LOC100910788; LOC501317; LOC684892; LOC100909462; LOC689635; LOC100909409; LOC100911002; LOC100910163; LOC100911516; RGD1561986; rno-mir-3584
17805031	7.26	7.19	1.05	0.0703893	0.039298	0.999562	Mxra8
17805459	6.42	6.34	1.05	0.0703893	0.041213	0.999562	Adhfe1
17794925	9.04	8.97	1.05	0.0703893	0.042177	0.999562	Cops7a
17664022	9.44	9.37	1.05	0.0703893	0.042688	0.999562	Thoc4
17623800	8.12	8.05	1.05	0.0703893	0.044093	0.999562	LOC690349
17858326	10.22	10.15	1.05	0.0703893	0.045332	0.999562	Plekhb2
17640286	9.89	9.82	1.05	0.0703893	0.046051	0.999562	Polr2g
17816275	9.18	9.11	1.05	0.0703893	0.046109	0.999562	Arf6
17718168	10.93	10.86	1.05	0.0703893	0.048408	0.999562	Prelid1
17646320	6.96	6.91	1.04	0.0565835	0.000849	0.999562	Jmjd4
17774581	9.03	8.97	1.04	0.0565835	0.004526	0.999562	Mdk
17648002	8.72	8.65	1.04	0.0565835	0.008921	0.999562	Txndc17
17801885	9.04	8.99	1.04	0.0565835	0.025576	0.999562	Rragc
17764003	8.66	8.6	1.04	0.0565835	0.030943	0.999562	Arfgap2
17717913	8.9	8.85	1.04	0.0565835	0.038976	0.999562	Naa35
17724936	11.72	11.66	1.04	0.0565835	0.042052	0.999562	Rpl39; LOC100361661; LOC100363391; LOC100364645
17814018	9.61	9.55	1.04	0.0565835	0.042138	0.999562	Fam98a
17731611	10.66	10.62	1.03	0.0426443	0.003722	0.999562	Arl2bp
17732480	8.83	8.79	1.03	0.0426443	0.006108	0.999562	Farsa
17842051	11.07	11.02	1.03	0.0426443	0.006545	0.999562	Sesn3
17716548	10.72	10.68	1.03	0.0426443	0.007526	0.999562	Zmynd11
17793404	8.76	8.72	1.03	0.0426443	0.013142	0.999562	Magi1
17642377	8.31	8.26	1.03	0.0426443	0.020064	0.999562	Mrpl43
17792158	8.57	8.53	1.03	0.0426443	0.023978	0.999562	Kdm3a
17802092	8.51	8.46	1.03	0.0426443	0.024552	0.999562	Oscp1
17854929	9.27	9.23	1.03	0.0426443	0.024864	0.999562	Armc8
17803776	9.98	9.93	1.03	0.0426443	0.045793	0.999562	Sdhb
17658951	9.26	9.21	1.03	0.0426443	0.047851	0.999562	Tnfaip1
17724896	9.09	9.06	1.02	0.0285692	0.002662	0.999562	Zfp521
17838957	9.76	9.72	1.02	0.0285692	0.010682	0.999562	Eif3d
17721440	10.24	10.21	1.02	0.0285692	0.049375	0.999562	Mib1
17810207	10.05	10.08	-1.02	-0.028569	0.032914	0.999562	Smap2
17820928	9.4	9.44	-1.03	-0.042644	0.049381	0.999562	Efr3b
17700110	9.37	9.42	-1.04	-0.056584	0.00267	0.999562	Zc3h13
17830667	10.39	10.45	-1.04	-0.056584	0.006027	0.999562	Tomm22
17821097	10.71	10.76	-1.04	-0.056584	0.013301	0.999562	Ddx1
17702184	9.02	9.07	-1.04	-0.056584	0.02564	0.999562	Rab2b
17808587	10.13	10.19	-1.04	-0.056584	0.030297	0.999562	Elavl2
17722731	7.91	7.97	-1.04	-0.056584	0.032173	0.999562	PCDHGA6
17639197	9.11	9.17	-1.04	-0.056584	0.033342	0.999562	Ankrd13d
17620723	7.07	7.15	-1.05	-0.070389	0.013757	0.999562	Hirip3
17687394	9.55	9.62	-1.05	-0.070389	0.018158	0.999562	Mark1
17643588	9.49	9.55	-1.05	-0.070389	0.019887	0.999562	Dexi
17761716	7.53	7.6	-1.05	-0.070389	0.023319	0.999562	Avpi1

Transcript Cluster ID	Injury Bi-weight Avg Signal (log2)	Control Bi-weight Avg Signal (log2)	Fold Change (linear) (Injury vs. Control)	Fold Change (log2)	ANOVA p-value (Injury vs. Control)	FDR p-value (Injury vs. Control)	Gene Symbol
17792187	8.83	8.9	-1.05	-0.070389	0.024514	0.999562	Mrpl35
17854075	4.2	4.28	-1.05	-0.070389	0.027798	0.999562	Fam83b
17746315	9.75	9.82	-1.05	-0.070389	0.043602	0.999562	Ralyl
17855786	9.79	9.85	-1.05	-0.070389	0.047228	0.999562	Wdr6
17794781	9.47	9.53	-1.05	-0.070389	0.047281	0.999562	Grcc10; LOC100911713
17822701	4.92	5.01	-1.06	-0.084064	0.001939	0.999562	Esr2
17619466	7.76	7.84	-1.06	-0.084064	0.009313	0.999562	Akip1
17713314	5.89	5.97	-1.06	-0.084064	0.010967	0.999562	Gdf2
17824403	9.46	9.54	-1.06	-0.084064	0.026901	0.999562	Cdc42bpb
17770545	5.66	5.74	-1.06	-0.084064	0.02791	0.999562	C030048H21Rik
17804603	9.2	9.28	-1.06	-0.084064	0.031152	0.999562	Chd5
17828097	8.34	8.43	-1.06	-0.084064	0.031378	0.999562	Csrp2
17704875	6.62	6.7	-1.06	-0.084064	0.032453	0.999562	LOC306096
17839278	7.22	7.31	-1.06	-0.084064	0.038685	0.999562	Rps19bp1
17610464	9.08	9.17	-1.06	-0.084064	0.039027	0.999562	Shprh
17814921	7.7	7.78	-1.06	-0.084064	0.040436	0.999562	Nol10
17865896	8.2	8.29	-1.06	-0.084064	0.04134	0.999562	RGD1561963
17714533	9.58	9.67	-1.06	-0.084064	0.047027	0.999562	Gprin1
17740644	8.7	8.79	-1.06	-0.084064	0.048664	0.999562	Tdrkh
17641203	9.49	9.59	-1.07	-0.097611	0.000513	0.897373	Smc5
17870953	7.87	7.98	-1.07	-0.097611	0.011808	0.999562	Bcor
17682462	7.79	7.89	-1.07	-0.097611	0.012102	0.999562	Kif26b
17788981	9.19	9.28	-1.07	-0.097611	0.017234	0.999562	Cacna2d1
17804509	9.28	9.38	-1.07	-0.097611	0.017842	0.999562	Nol9
17805774	7.28	7.38	-1.07	-0.097611	0.01905	0.999562	Plekhf2
17773262	5.83	5.93	-1.07	-0.097611	0.02092	0.999562	Chrna1
17755510	9.86	9.96	-1.07	-0.097611	0.020969	0.999562	Rev3l
17627906	10.97	11.07	-1.07	-0.097611	0.024701	0.999562	LOC100363248
17643106	9.04	9.14	-1.07	-0.097611	0.03127	0.999562	Hspa12a
17881809	8.97	9.08	-1.07	-0.097611	0.034708	0.999562	LOC688318; Cyp27b1; Chchd2; LOC684871; Fabp5; Lce1l; Lsm2; LOC100362916; LOC682812; LOC684372; LOC100366231; LOC682352; LOC100910788; LOC501317; LOC684892; LOC100909462; LOC689635; LOC100909409; LOC100911002; LOC100910163; LOC100911516; RGD1561986; rno-mir-3584
17793817	8.5	8.6	-1.07	-0.097611	0.038465	0.999562	Tada3; Tada3lb
17787596	3.11	3.21	-1.07	-0.097611	0.040609	0.999562	Klre1
17770144	7.11	7.21	-1.07	-0.097611	0.045419	0.999562	Tor4a
17661681	6.17	6.27	-1.07	-0.097611	0.045567	0.999562	Brca1
17718262	5.97	6.06	-1.07	-0.097611	0.04782	0.999562	Eif4e1b
17688028	6.81	6.91	-1.07	-0.097611	0.049408	0.999562	Tgfbr3
17709173	6.9	7.02	-1.08	-0.111031	0.004014	0.999562	Erich1
17667934	8.16	8.27	-1.08	-0.111031	0.010728	0.999562	Gart
17872886	4.95	5.06	-1.08	-0.111031	0.015219	0.999562	Heph
17798570	8.54	8.66	-1.08	-0.111031	0.016173	0.999562	Tdrd7
17655641	9.54	9.65	-1.08	-0.111031	0.01842	0.999562	Lsm11
17690435	7.92	8.03	-1.08	-0.111031	0.022923	0.999562	Zfp518b
17730641	8.32	8.43	-1.08	-0.111031	0.026101	0.999562	Gse1
17882193	6	6.11	-1.08	-0.111031	0.02705	0.999562	LOC688318; Cyp27b1; Chchd2; LOC684871; Fabp5; Lce1l; Lsm2; LOC100362916; LOC682812; LOC684372; LOC100366231; LOC682352; LOC100910788; LOC501317; LOC684892; LOC100909462; LOC689635; LOC100909409; LOC100911002; LOC100910163; LOC100911516; RGD1561986; rno-mir-3584
17853371	4.65	4.77	-1.08	-0.111031	0.029428	0.999562	Iqch
17733304	9.93	10.05	-1.08	-0.111031	0.031098	0.999562	Smpd3
17777757	7.03	7.14	-1.08	-0.111031	0.033124	0.999562	Kif16b
17881547	6.67	6.78	-1.08	-0.111031	0.033357	0.999562	LOC688318; Cyp27b1; Chchd2; LOC684871; Fabp5; Lce1l; Lsm2; LOC100362916; LOC682812; LOC684372; LOC100366231; LOC682352; LOC100910788; LOC501317; LOC684892; LOC100909462; LOC689635; LOC100909409; LOC100911002; LOC100910163; LOC100911516; RGD1561986; rno-mir-3584

Transcript Cluster ID	Injury Bi-weight Avg Signal (log2)	Control Bi-weight Avg Signal (log2)	Fold Change (linear) (Injury vs. Control)	Fold Change (log2)	ANOVA p-value (Injury vs. Control)	FDR p-value (Injury vs. Control)	Gene Symbol
17729655	8.27	8.37	-1.08	-0.111031	0.035852	0.999562	Slc7a6
17660607	8.33	8.44	-1.08	-0.111031	0.036198	0.999562	Pcgf2; LOC681958
17622317	8.03	8.14	-1.08	-0.111031	0.037541	0.999562	Tspan4; LOC100911766
17881825	6.72	6.83	-1.08	-0.111031	0.038424	0.999562	LOC688318; Cyp27b1; Chchd2; LOC684871; Fabp5; Lce1l; Lsm2; LOC100362916; LOC682812; LOC684372; LOC100366231; LOC682352; LOC100910788; LOC501317; LOC684892; LOC100909462; LOC689635; LOC100909409; LOC100911002; LOC100910163; LOC100911516; RGD1561986; rno-mir-3584
17667162	9.57	9.68	-1.08	-0.111031	0.040787	0.999562	Pi4ka
17653311	9.45	9.56	-1.08	-0.111031	0.048538	0.999562	Hgs
17739281	6.15	6.26	-1.08	-0.111031	0.049203	0.999562	Fam198b
17861093	8.62	8.75	-1.09	-0.124328	0.001667	0.999562	Dis3l2
17802419	6.67	6.79	-1.09	-0.124328	0.007526	0.999562	Sync
17738403	8.51	8.63	-1.09	-0.124328	0.012895	0.999562	Ccrn4l
17801737	6.75	6.88	-1.09	-0.124328	0.013568	0.999562	Cited4
17833575	8.84	8.96	-1.09	-0.124328	0.017975	0.999562	Sppl2b
17695125	8.31	8.43	-1.09	-0.124328	0.018517	0.999562	Jakmip1
17692728	8.12	8.24	-1.09	-0.124328	0.019542	0.999562	Lrrc8d
17812744	7.91	8.03	-1.09	-0.124328	0.021935	0.999562	Pex14
17731019	8.38	8.51	-1.09	-0.124328	0.025528	0.999562	Gas8
17632953	6.93	7.05	-1.09	-0.124328	0.025622	0.999562	Spaca4
17838221	5.67	5.79	-1.09	-0.124328	0.02968	0.999562	Ly6c; LOC100911104; LOC100912078
17799518	5.1	5.23	-1.09	-0.124328	0.034083	0.999562	Tyrp1
17715650	8.68	8.81	-1.09	-0.124328	0.042189	0.999562	Lrrc16a
17856014	6.39	6.51	-1.09	-0.124328	0.04294	0.999562	Pth1r
17861509	5.6	5.72	-1.09	-0.124328	0.045701	0.999562	Mlph
17651061	5.36	5.49	-1.09	-0.124328	0.046516	0.999562	Klhl10
17882457	5.89	6.02	-1.09	-0.124328	0.049998	0.999562	LOC688318; Cyp27b1; Chchd2; LOC684871; Fabp5; Lce1l; Lsm2; LOC100362916; LOC682812; LOC684372; LOC100366231; LOC682352; LOC100910788; LOC501317; LOC684892; LOC100909462; LOC689635; LOC100909409; LOC100911002; LOC100910163; LOC100911516; RGD1561986; rno-mir-3584
17762887	9.25	9.38	-1.1	-0.137504	0.003042	0.999562	Dlx1
17818030	9.35	9.49	-1.1	-0.137504	0.004406	0.999562	Unc79
17706586	7.19	7.33	-1.1	-0.137504	0.007469	0.999562	Grid1
17708097	3.85	3.99	-1.1	-0.137504	0.009306	0.999562	Fgl1
17625649	4.89	5.02	-1.1	-0.137504	0.011141	0.999562	Hpse2
17716754	3.55	3.68	-1.1	-0.137504	0.012853	0.999562	Akr1c3
17841442	5.27	5.41	-1.1	-0.137504	0.013116	0.999562	Krt78
17765197	7.98	8.12	-1.1	-0.137504	0.015925	0.999562	Mapkbp1
17832737	4.33	4.46	-1.1	-0.137504	0.01866	0.999562	Hoxc5
17878135	5.62	5.76	-1.1	-0.137504	0.021004	0.999562	Cxcr3
17827384	7.46	7.59	-1.1	-0.137504	0.021058	0.999562	Nup37
17799278	7.42	7.55	-1.1	-0.137504	0.026823	0.999562	Col27a1
17723564	6.2	6.33	-1.1	-0.137504	0.027822	0.999562	Cd74
17745609	4.39	4.53	-1.1	-0.137504	0.032004	0.999562	Card6
17839341	3.6	3.74	-1.1	-0.137504	0.036287	0.999562	Dnajb7
17850063	8.25	8.4	-1.1	-0.137504	0.040307	0.999562	Zfp426
17821455	5.07	5.21	-1.1	-0.137504	0.041174	0.999562	Slc26a4
17627542	6.59	6.73	-1.1	-0.137504	0.045065	0.999562	RGD1560020_predicted
17627955	8.6	8.75	-1.11	-0.15056	0.000531	0.897373	Brd9
17852970	8.81	8.96	-1.11	-0.15056	0.010686	0.999562	Csk
17652634	7.23	7.39	-1.11	-0.15056	0.013105	0.999562	Armc7
17720573	6.93	7.08	-1.11	-0.15056	0.013174	0.999562	Ankrd16
17765071	6.05	6.2	-1.11	-0.15056	0.014601	0.999562	Dll4
17626143	7.42	7.57	-1.11	-0.15056	0.01989	0.999562	Fbxl15
17790424	9.49	9.64	-1.11	-0.15056	0.020733	0.999562	Plxna4a
17864208	6.71	6.86	-1.11	-0.15056	0.022111	0.999562	Tex30
17856061	8.11	8.25	-1.11	-0.15056	0.023442	0.999562	Mlh1
17635910	4.79	4.94	-1.11	-0.15056	0.028572	0.999562	Olr208
17797076	9.99	10.15	-1.11	-0.15056	0.035806	0.999562	Xkr4
17812762	8.01	8.15	-1.11	-0.15056	0.036652	0.999562	Apitd1; Cort; Kif1b; LOC100360180

Transcript Cluster ID	Injury Bi-weight Avg Signal (log2)	Control Bi-weight Avg Signal (log2)	Fold Change (linear) (Injury vs. Control)	Fold Change (log2)	ANOVA p-value (Injury vs. Control)	FDR p-value (Injury vs. Control)	Gene Symbol
17712212	4	4.15	-1.11	-0.15056	0.037713	0.999562	Poteg
17635598	4.22	4.37	-1.11	-0.15056	0.042784	0.999562	Olr128
17722664	5.55	5.69	-1.11	-0.15056	0.047349	0.999562	Pcdhb11
17667134	7.77	7.94	-1.12	-0.163499	0.007439	0.999562	Slc7a4
17646195	5.51	5.68	-1.12	-0.163499	0.008873	0.999562	Olr1437; LOC100911223
17770130	5.89	6.06	-1.12	-0.163499	0.015396	0.999562	Noxa1
17827749	8.39	8.55	-1.12	-0.163499	0.015486	0.999562	Eea1
17767857	6.43	6.6	-1.12	-0.163499	0.025224	0.999562	RGD1561517
17882529	6.29	6.45	-1.12	-0.163499	0.027223	0.999562	LOC688318; Cyp27b1; Chchd2; LOC684871; Fabp5; Lce1l; Lsm2; LOC100362916; LOC682812; LOC684372; LOC100366231; LOC682352; LOC100910788; LOC501317; LOC684892; LOC100909462; LOC689635; LOC100909409; LOC100911002; LOC100910163; LOC100911516; RGD1561986; rno-mir-3584
17722661	7.66	7.83	-1.12	-0.163499	0.0291	0.999562	Pcdhb10
17825882	3.64	3.8	-1.12	-0.163499	0.030151	0.999562	Olr956
17650508	4.77	4.94	-1.12	-0.163499	0.030349	0.999562	Hoxb13
17622552	4.72	4.89	-1.12	-0.163499	0.032663	0.999562	Slc22a18
17882243	6.09	6.25	-1.12	-0.163499	0.032783	0.999562	LOC688318; Cyp27b1; Chchd2; LOC684871; Fabp5; Lce1l; Lsm2; LOC100362916; LOC682812; LOC684372; LOC100366231; LOC682352; LOC100910788; LOC501317; LOC684892; LOC100909462; LOC689635; LOC100909409; LOC100911002; LOC100910163; LOC100911516; RGD1561986; rno-mir-3584
17677600	7.54	7.7	-1.12	-0.163499	0.037361	0.999562	Snrnp35
17791731	4.17	4.33	-1.12	-0.163499	0.0394	0.999562	Vom1r89
17882479	7.97	8.14	-1.12	-0.163499	0.042896	0.999562	LOC688318; Cyp27b1; Chchd2; LOC684871; Fabp5; Lce1l; Lsm2; LOC100362916; LOC682812; LOC684372; LOC100366231; LOC682352; LOC100910788; LOC501317; LOC684892; LOC100909462; LOC689635; LOC100909409; LOC100911002; LOC100910163; LOC100911516; RGD1561986; rno-mir-3584
17753559	5.28	5.44	-1.12	-0.163499	0.044233	0.999562	Armc12
17789837	5.45	5.63	-1.13	-0.176323	0.006861	0.999562	Tfec
17881501	4.38	4.56	-1.13	-0.176323	0.009414	0.999562	LOC688318; Cyp27b1; Chchd2; LOC684871; Fabp5; Lce1l; Lsm2; LOC100362916; LOC682812; LOC684372; LOC100366231; LOC682352; LOC100910788; LOC501317; LOC684892; LOC100909462; LOC689635; LOC100909409; LOC100911002; LOC100910163; LOC100911516; RGD1561986; rno-mir-3584
17737321	6.28	6.46	-1.13	-0.176323	0.009711	0.999562	Mir124-2; rno-mir-124-2
17789975	6.38	6.56	-1.13	-0.176323	0.013234	0.999562	Fezf1
17752741	5.48	5.67	-1.13	-0.176323	0.013776	0.999562	Lta
17881975	6.81	6.98	-1.13	-0.176323	0.014017	0.999562	LOC688318; Cyp27b1; Chchd2; LOC684871; Fabp5; Lce1l; Lsm2; LOC100362916; LOC682812; LOC684372; LOC100366231; LOC682352; LOC100910788; LOC501317; LOC684892; LOC100909462; LOC689635; LOC100909409; LOC100911002; LOC100910163; LOC100911516; RGD1561986; rno-mir-3584
17798739	7.58	7.75	-1.13	-0.176323	0.02002	0.999562	Stx17
17627579	3.33	3.5	-1.13	-0.176323	0.021155	0.999562	RGD1560849
17723683	6.23	6.41	-1.13	-0.176323	0.024079	0.999562	Il17b
17748210	3.7	3.87	-1.13	-0.176323	0.026053	0.999562	Tdo2
17699381	8.4	8.58	-1.13	-0.176323	0.030296	0.999562	Shisa2
17818335	6.05	6.22	-1.13	-0.176323	0.039344	0.999562	Mir342; rno-mir-342
17760924	7.16	7.34	-1.13	-0.176323	0.047303	0.999562	Endog
17613652	8.94	9.12	-1.14	-0.189034	0.011531	0.999562	Tmem160
17702684	7.74	7.93	-1.14	-0.189034	0.012452	0.999562	Cdh24
17623111	8.09	8.28	-1.14	-0.189034	0.013732	0.999562	Bles03
17652713	6.7	6.89	-1.14	-0.189034	0.01893	0.999562	Tsen54
17847663	5.24	5.43	-1.14	-0.189034	0.019377	0.999562	Mst1

Transcript Cluster ID	Injury Bi-weight Avg Signal (log2)	Control Bi-weight Avg Signal (log2)	Fold Change (linear) (Injury vs. Control)	Fold Change (log2)	ANOVA p-value (Injury vs. Control)	FDR p-value (Injury vs. Control)	Gene Symbol
17675782	7.86	8.05	-1.14	-0.189034	0.021666	0.999562	Zkscan5
17675570	5.67	5.86	-1.14	-0.189034	0.025101	0.999562	Tex26
17613711	7.59	7.78	-1.14	-0.189034	0.036669	0.999562	Prkd2
17873839	6.74	6.93	-1.14	-0.189034	0.037632	0.999562	Cenpi
17640763	5.04	5.22	-1.14	-0.189034	0.038486	0.999562	Plac1l
17826222	7.76	7.94	-1.14	-0.189034	0.044327	0.999562	Lingo3
17624335	3.46	3.66	-1.15	-0.201634	0.002856	0.999562	Olr348
17737825	8	8.2	-1.15	-0.201634	0.006105	0.999562	LOC100912162
17848680	6.87	7.07	-1.15	-0.201634	0.011533	0.999562	Ctdspl
17730478	7.37	7.57	-1.15	-0.201634	0.011628	0.999562	LOC690350
17880257	5.89	6.09	-1.15	-0.201634	0.014659	0.999562	RGD1560412
17726365	6.4	6.61	-1.15	-0.201634	0.014907	0.999562	Fbn2
17670074	4.77	4.97	-1.15	-0.201634	0.015092	0.999562	Rtp4
17801650	6.5	6.7	-1.15	-0.201634	0.019436	0.999562	Zmynd12
17848419	5.02	5.23	-1.15	-0.201634	0.024628	0.999562	Dclk3
17742029	6.86	7.06	-1.15	-0.201634	0.028089	0.999562	Vav3
17752416	7.63	7.83	-1.15	-0.201634	0.03441	0.999562	Rpp21
17646399	7.54	7.74	-1.15	-0.201634	0.039762	0.999562	Lrrc48
17822823	9.44	9.64	-1.15	-0.201634	0.045633	0.999562	Tmem229b
17615098	5.82	6.04	-1.16	-0.214125	0.010308	0.999562	Ggn
17617432	6.94	7.16	-1.16	-0.214125	0.014224	0.999562	Ttc23
17760074	5	5.22	-1.16	-0.214125	0.021963	0.999562	RGD1564133
17810254	4.19	4.4	-1.16	-0.214125	0.023917	0.999562	RGD1560700
17780256	4.67	4.89	-1.16	-0.214125	0.024143	0.999562	RGD1306208
17882725	4.66	4.87	-1.16	-0.214125	0.028592	0.999562	LOC688318; Cyp27b1; Chchd2; LOC684871; Fabp5; Lce1l; Lsm2; LOC100362916; LOC682812; LOC684372; LOC100366231; LOC682352; LOC100910788; LOC501317; LOC684892; LOC100909462; LOC689635; LOC100909409; LOC100911002; LOC100910163; LOC100911516; RGD1561986; rno-mir-3584
17764262	4.92	5.14	-1.16	-0.214125	0.034809	0.999562	Rag2
17633143	8.4	8.6	-1.16	-0.214125	0.040273	0.999562	Sergef
17657858	4.4	4.61	-1.16	-0.214125	0.040368	0.999562	Slc35g3
17764628	6.62	6.84	-1.17	-0.226509	0.002342	0.999562	RGD1566059
17762269	3.96	4.18	-1.17	-0.226509	0.011782	0.999562	Galnt5
17866307	5.65	5.88	-1.17	-0.226509	0.016164	0.999562	Dnajb3
17781980	5.77	5.99	-1.17	-0.226509	0.046164	0.999562	Fam71f1
17832722	3.86	4.08	-1.17	-0.226509	0.04936	0.999562	Mir196a; rno-mir-196a
17646585	6.01	6.25	-1.18	-0.238787	0.000097	0.832906	Aldh3a1
17878255	5.36	5.6	-1.18	-0.238787	0.01273	0.999562	RGD1564124
17647306	5.7	5.94	-1.18	-0.238787	0.015396	0.999562	Slc25a35
17630465	5.47	5.71	-1.18	-0.238787	0.018793	0.999562	Tescl
17879619	3.28	3.52	-1.18	-0.238787	0.021179	0.999562	Mir743b; rno-mir-743b
17657398	7.44	7.68	-1.18	-0.238787	0.025488	0.999562	Ntn1
17787649	3.46	3.7	-1.18	-0.238787	0.029019	0.999562	Tas2r145
17778164	5.11	5.36	-1.18	-0.238787	0.0294	0.999562	Vsx1
17850348	6.78	7.02	-1.18	-0.238787	0.03161	0.999562	Spc24
17755055	5.44	5.67	-1.18	-0.238787	0.038644	0.999562	Neurog3
17838768	6.03	6.27	-1.18	-0.238787	0.040682	0.999562	Foxh1
17611264	5.86	6.1	-1.19	-0.250962	0.007457	0.999562	Zfp72
17640353	4.01	4.26	-1.19	-0.250962	0.013533	0.999562	LOC686411
17883147	6.96	7.21	-1.19	-0.250962	0.019198	0.999562	LOC688318; Cyp27b1; Chchd2; LOC684871; Fabp5; Lce1l; Lsm2; LOC100362916; LOC682812; LOC684372; LOC100366231; LOC682352; LOC100910788; LOC501317; LOC684892; LOC100909462; LOC689635; LOC100909409; LOC100911002; LOC100910163; LOC100911516; RGD1561986; rno-mir-3584
17702025	4.96	5.22	-1.19	-0.250962	0.022155	0.999562	Rnase9
17709283	3.99	4.25	-1.19	-0.250962	0.029894	0.999562	Adprhl1; Grtp1
17638165	5.92	6.17	-1.19	-0.250962	0.030342	0.999562	Olr288
17723575	5.22	5.47	-1.19	-0.250962	0.030495	0.999562	Arsi
17872922	4.64	4.89	-1.19	-0.250962	0.045879	0.999562	Pgr15l
17613764	5.13	5.38	-1.19	-0.250962	0.046158	0.999562	Cgm4
17618416	7.09	7.35	-1.19	-0.250962	0.048962	0.999562	RGD1562118

Transcript Cluster ID	Injury Bi-weight Avg Signal (log2)	Control Bi-weight Avg Signal (log2)	Fold Change (linear) (Injury vs. Control)	Fold Change (log2)	ANOVA p-value (Injury vs. Control)	FDR p-value (Injury vs. Control)	Gene Symbol
17662434	5.43	5.69	-1.2	-0.263034	0.003906	0.999562	Gh1
17695587	5.19	5.46	-1.2	-0.263034	0.010222	0.999562	Slc5a1
17806341	5.48	5.74	-1.2	-0.263034	0.019079	0.999562	RGD1563056
17746204	2.61	2.87	-1.2	-0.263034	0.019131	0.999562	Sirpb2
17837374	6.01	6.27	-1.2	-0.263034	0.037413	0.999562	Dscc1
17720596	4.74	5.02	-1.21	-0.275007	0.030947	0.999562	Il2ra
17806775	5	5.28	-1.21	-0.275007	0.04279	0.999562	Cd72
17613011	7.12	7.39	-1.21	-0.275007	0.049261	0.999562	Zfp579
17640644	5.97	6.26	-1.22	-0.286881	0.001559	0.999562	Ms4a8
17770759	7.7	7.99	-1.22	-0.286881	0.014427	0.999562	Abo
17658394	6.24	6.52	-1.22	-0.286881	0.01755	0.999562	Smtnl2
17649345	6.36	6.65	-1.22	-0.286881	0.018717	0.999562	Rnf135
17703435	3.77	4.06	-1.22	-0.286881	0.027413	0.999562	RGD1563302
17871605	5.31	5.59	-1.22	-0.286881	0.028331	0.999562	LOC681355; LOC683430
17645229	7.77	8.05	-1.22	-0.286881	0.034384	0.999562	Fndc9
17662478	5.11	5.4	-1.22	-0.286881	0.046112	0.999562	Icam2
17789491	4.11	4.42	-1.23	-0.298658	0.016281	0.999562	Hepacam2
17624498	5.16	5.45	-1.23	-0.298658	0.023495	0.999562	Aldh1a1
17881819	4.97	5.28	-1.23	-0.298658	0.036618	0.999562	LOC688318; Cyp27b1; Chchd2; LOC684871; Fabp5; Lce1l; Lsm2; LOC100362916; LOC682812; LOC684372; LOC100366231; LOC682352; LOC100910788; LOC501317; LOC684892; LOC100909462; LOC689635; LOC100909409; LOC100911002; LOC100910163; LOC100911516; RGD1561986; rno-mir-3584
17613328	3.55	3.85	-1.24	-0.31034	0.020359	0.999562	Vom1r51; LOC100364634
17642959	5.34	5.65	-1.24	-0.31034	0.026502	0.999562	Nrap
17846461	5.1	5.43	-1.25	-0.321928	0.000188	0.876358	Trim43a
17632333	5.17	5.51	-1.26	-0.333424	0.015071	0.999562	Klk1c2
17834465	5.16	5.49	-1.26	-0.333424	0.026185	0.999562	RGD1564285
17635918	5.3	5.63	-1.26	-0.333424	0.036144	0.999562	Olr217
17881481	2.37	2.7	-1.26	-0.333424	0.038171	0.999562	LOC688318; Cyp27b1; Chchd2; LOC684871; Fabp5; Lce1l; Lsm2; LOC100362916; LOC682812; LOC684372; LOC100366231; LOC682352; LOC100910788; LOC501317; LOC684892; LOC100909462; LOC689635; LOC100909409; LOC100911002; LOC100910163; LOC100911516; RGD1561986; rno-mir-3584
17829999	6.66	7	-1.27	-0.344828	0.001368	0.999562	Tigd5
17855604	3.85	4.2	-1.27	-0.344828	0.008708	0.999562	Gnat1
17656467	2.64	2.99	-1.27	-0.344828	0.018523	0.999562	Olr1418
17855866	5.69	6.03	-1.27	-0.344828	0.033134	0.999562	Camp
17807275	2.14	2.48	-1.27	-0.344828	0.036952	0.999562	Olr850
17613125	5.51	5.87	-1.28	-0.356144	0.01924	0.999562	Tmem86b
17881545	6.35	6.71	-1.28	-0.356144	0.033797	0.999562	LOC688318; Cyp27b1; Chchd2; LOC684871; Fabp5; Lce1l; Lsm2; LOC100362916; LOC682812; LOC684372; LOC100366231; LOC682352; LOC100910788; LOC501317; LOC684892; LOC100909462; LOC689635; LOC100909409; LOC100911002; LOC100910163; LOC100911516; RGD1561986; rno-mir-3584
17664603	5.7	6.06	-1.28	-0.356144	0.038711	0.999562	Mrap
17805286	3.37	3.74	-1.29	-0.367371	0.011564	0.999562	Xkr9
17764646	4.14	4.51	-1.29	-0.367371	0.023199	0.999562	Olr786; Olr796; LOC100909613
17843409	3.57	3.94	-1.29	-0.367371	0.04919	0.999562	Olr1329
17614445	7.34	7.71	-1.3	-0.378512	0.01824	0.999562	4732471J01Rik
17818691	6.75	7.13	-1.3	-0.378512	0.025911	0.999562	Mir3576; Mir539; rno-mir-3576; rno-mir-539
17787401	5.38	5.75	-1.3	-0.378512	0.037561	0.999562	Akap3
17775517	5.11	5.51	-1.31	-0.389567	0.022797	0.999562	Actc1; Acta2
17874079	4.3	4.69	-1.31	-0.389567	0.030096	0.999562	Il1rapl2
17841759	2.53	2.93	-1.32	-0.400538	0.001612	0.999562	LOC681366
17880505	3.51	3.91	-1.32	-0.400538	0.019368	0.999562	RGD1565369
17613324	2.41	2.82	-1.33	-0.411426	0.012008	0.999562	Vom1r49
17850002	3.79	4.2	-1.33	-0.411426	0.032095	0.999562	Olr1137; LOC100911893
17763627	1.7	2.1	-1.33	-0.411426	0.042695	0.999562	Olr526; Olr532
17687607	4.9	5.33	-1.34	-0.422233	0.014428	0.999562	Fam71a

Transcript Cluster ID	Injury Bi-weight Avg Signal (log2)	Control Bi-weight Avg Signal (log2)	Fold Change (linear) (Injury vs. Control)	Fold Change (log2)	ANOVA p-value (Injury vs. Control)	FDR p-value (Injury vs. Control)	Gene Symbol
17798915	4.87	5.29	-1.34	-0.422233	0.014541	0.999562	Olr848
17876439	5.54	5.96	-1.34	-0.422233	0.037594	0.999562	Mycs
17635936	3.55	3.97	-1.34	-0.422233	0.047519	0.999562	Olr232
17834268	1.91	2.35	-1.35	-0.432959	0.019018	0.999562	LOC687068
17619051	3.34	3.78	-1.35	-0.432959	0.027538	0.999562	Olr53
17851138	2.95	3.38	-1.35	-0.432959	0.032132	0.999562	Olr1195
17785395	2.18	2.61	-1.35	-0.432959	0.036689	0.999562	Vom1r100
17679465	3.44	3.89	-1.37	-0.454176	0.00779	0.999562	Serpinb7
17728532	3.67	4.13	-1.37	-0.454176	0.010372	0.999562	LOC367516
17842229	3.48	3.94	-1.37	-0.454176	0.019451	0.999562	Olr1156
17875457	5.25	5.71	-1.38	-0.464668	0.010359	0.999562	Rhox8; Rhox7
17619038	4.91	5.37	-1.38	-0.464668	0.022547	0.999562	Olr46
17878662	5.62	6.09	-1.38	-0.464668	0.025557	0.999562	RGD1561582
17774387	2.92	3.39	-1.39	-0.475085	0.009096	0.999562	Olr735
17619208	2.15	2.62	-1.39	-0.475085	0.027474	0.999562	Olr189
17805665	3.88	4.36	-1.4	-0.485427	0.005972	0.999562	Cyp7a1
17623944	4.49	4.97	-1.4	-0.485427	0.014898	0.999562	Scgb2a2
17612847	5.66	6.15	-1.41	-0.495695	0.046668	0.999562	Vom2r-ps45
17774158	2.91	3.43	-1.43	-0.516015	0.000114	0.832906	Olr510
17650210	5.34	5.9	-1.47	-0.555816	0.029316	0.999562	LOC100363423
17661722	5.31	5.87	-1.48	-0.565597	0.046818	0.999562	Meox1
17842868	4.67	5.36	-1.61	-0.687061	0.011343	0.999562	LOC100365695
17813579	3.56	4.24	-1.61	-0.687061	0.026214	0.999562	4921513D11Rik
17635975	2.89	3.61	-1.64	-0.713696	0.020443	0.999562	Olr239
17774178	4.23	5.01	-1.72	-0.782409	0.040001	0.999562	Olr531
17640209	2.31	3.22	-1.88	-0.910733	0.009028	0.999562	UST4r; Ust5r
17749701	3.42	4.46	-2.05	-1.035624	0.0298	0.999562	Olr390

Supplementary **Table 3:** Cell-cell adhesion transcripts changed 24 h after injury (p < 0.1).

Transcript Cluster ID	Injury Bi-weight Avg Signal (log2)	Control Bi-weight Avg Signal (log2)	Fold Change (linear) (Injury vs. Control)	Fold Change (log2)	ANOVA p-value (Injury vs. Control)	FDR p-value (Injury vs. Control)	Gene Symbol
17812245	9.98	9.76	1.16	0.2141248	0.004167	0.999562	Pdpn
17682462	7.79	7.89	-1.07	-0.097611	0.012102	0.999562	Kif26b
17702684	7.74	7.93	-1.14	-0.189034	0.012452	0.999562	Cdh24
17736736	8.23	8.14	1.07	0.0976108	0.014993	0.999562	Myo10
17657398	7.44	7.68	-1.18	-0.238787	0.025488	0.999562	Ntn1
17662478	5.11	5.4	-1.22	-0.286881	0.046112	0.999562	Icam2
17779108	6	5.83	1.13	0.1763228	0.050883	0.999562	Ada
17630418	6.55	6.28	1.2	0.2630344	0.054165	0.999562	PVR
17849247	4.13	3.99	1.1	0.1375035	0.066859	0.999562	Ccr5
17756618	8.05	8.11	-1.04	-0.056584	0.069545	0.999562	Tnxb
17799844	5.12	5.39	-1.2	-0.263034	0.081691	0.999562	Tek
17777980	6.16	5.81	1.27	0.3448285	0.089568	0.999562	Cd93
17732686	10.09	10.18	-1.06	-0.084064	0.093862	0.999562	Lphn1
17711838	9.15	9.19	-1.03	-0.042644	0.095813	0.999562	Fat1
